# Two Cases of Traumatic Tetanus

**Published:** 1885-07-20

**Authors:** H. Speir

**Affiliations:** Jordan, Minn.


### TWO CASES OF TRAUMATIC TETANUS.

By H. SPEIR, M. D., Jordan, Minn.

The cases of traumatic tetanus reported by Dr. Collins, in the *Lancet* of February 15, recall to my mind a couple of similar cases which I had to treat in quick succession:

### CASE I.

July 3d, 1882, Ole B., æt. 7; small wound on index finger of left hand, made by the explosion of a toy pistol. I cleaned the wound and applied a carbolic dressing. Everything progressed well till July 10, when I was called to his house and found the boy with trismus, general tetanus and opisthotonos. Used the various antispasmodics; amputated the finger, which was considerably swelled and inflamed; finally procured some extract of physostigma for hypodermic injection, but could not check the disease. Relief came with death on July 12. No foreign substance had been left in the wound.

### CASE II.

In the fall of 1882 was called one night to see Mrs. L., a young, healthy, vigorous woman, in consultation with Dr. Blekoe, now deceased. Found patient with well marked trismus, frequently returning general tonic convulsions, occasionally a slight degree of opisthotonos. In the course of the day her physician, absent at this time, had opened a felon on one of her fingers, but instead of making a good full incision, had only made a small, punctured wound. Having seen the uselessness of moderate doses in the previous case, we concluded to give remedies at once in large doses, and consequently gave her half an ounce of bromide of potassium. In the course of two hours we administered an ounce and a half of the drug. The spasms were then completely relaxed and did not return. In the morning the wound on the finger was enlarged and the case made a good recovery.

Writing entirely from memory, I cannot give any details, such as temperature, rate and quality of pulse, etc. I only wish to say that if such a case should ever come into my hands again I would not lose time by giving chloral or bromide in half drachm doses, but at once resort to such heroic doses as half an ounce of bromide. —*North Western Lancet.*

